# Surfactant-free ionic liquid-based nanofluids with remarkable thermal conductivity enhancement at very low loading of graphene

**DOI:** 10.1186/1556-276X-7-314

**Published:** 2012-06-19

**Authors:** Fuxian Wang, Lijuan Han, Zhengguo Zhang, Xiaoming Fang, Jingjing Shi, Wenshi Ma

**Affiliations:** 1 Key Laboratory of Enhanced Heat Transfer and Energy Conservation, the Ministry of Education, School of Chemistry and Chemical Engineering, South China University of Technology, Guangzhou, 510640, China; 2 School of Materials Science and Engineering, South China University of Technology, Guangzhou, 510640, China

**Keywords:** Ionanofluid, Graphene, MWCNTs, Thermal conductivity, Specific heat, Viscosity

## Abstract

We report for the first time the preparation of highly stable graphene (GE)-based nanofluids with ionic liquid as base fluids (ionic liquid-based nanofluids (Ionanofluids)) without any surfactant and the subsequent investigations on their thermal conductivity, specific heat, and viscosity. The microstructure of the GE and MWCNTs are observed by transmission electron microscope. Thermal conductivity (TC), specific heat, and viscosity of these Ionanofluids were measured for different weight fractions and at varying temperatures, demonstrating that the Ionanofluids exhibit considerably higher TC and lower viscosity than that of their base fluids without significant specific heat decrease. An enhancement in TC by about 15.5% and 18.6% has been achieved at 25 °C and 65 °C respectively for the GE-based nanofluid at mass fraction of as low as 0.06%, which is larger than that of the MWCNT-dispersed nanofluid at the same loading. When the temperature rises, the TC and specific heat of the Ionanofluid increase clearly, while the viscosity decreases sharply. Moreover, the viscosity of the prepared Ionanofluids is lower than that of the base fluid. All these advantages of this new kind of Ionanofluid make it an ideal fluid for heat transfer and thermal storage.

## Background

A nanofluid is a dilute suspension produced by dispersion of metallic or nonmetallic nanomaterials with a typical size of less than 100 nm in a base liquid, having the advantages of high dispersion stability and reduced pumping power and particle clogging as compared with conventional solid–liquid suspensions for heat transfer intensifications [1]. Since the pioneer work by Chol in 1995 [2], nanofluids have attracted extensive attention due to their enhanced thermophysical properties and heat transfer performance and their potential applications in many fields including cooling, thermal power generation, refrigeration, and so on [3]. Up to now, most of the previous researches have been focused on the nanofluids based on water, ethylene glycol, and synthetic oil [4-6]. Although these base fluids are readily available, water and ethylene glycol are usually used in relatively low temperature, and synthetic oil suffers from high vapor pressure and poor thermal stability. Therefore, it is necessary to develop novel nanofluids based on the fluids other than these conventional fluids.

Ionic liquids (ILs), organic salts with low melting points, have the characteristics of a wide range of liquid temperature, low vapor pressure, and high thermal stability, which make them possibly be used as a new group of heat transfer fluids for heat exchange in chemical plants, absorption cooling cycle system [7], and solar thermal power generation [8], where water and ethylene glycol may not be suitable for the application owing to the limitation of their thermophysical and chemical properties. Consequently, the nanofluids based on ILs are being explored intensely in recent years, in which Au [9], CuO [10], Al_2_O_3_[11], and multi-walled carbon nanotubes (MWCNTs) [12] have been used as the nanoadditive. It has been shown that the ionic liquid-based fluids (Ionanofluids) exhibit enhanced thermal conductivity (TC) as compared with the pure ILs, which just overcomes the inherent shortcoming of ILs. GE is a novel carbon nanomaterial with excellent electronic, mechanical, and thermal properties. The TC of GE is as large as around 5,000 W/m K, which makes it to be the most promising nanoadditive for nanofluids [13]. Accordingly, the nanofluids containing GE have attracted an increasing attention in the past 2 years, in which only the conventional fluids including water [14], ethylene glycol [15] and engine oil [16] have been used as the base fluids. In order to obtain stable GE-dispersed nanofluids, several measures have been taken in those previous work, such as adding surfactants into the nanofluids [15], making GE functionalized by chemical treatments [17], or using graphene oxide instead of GE as the additive [18]. It has been presented that GE can be functionalized by ILs through noncovalent interactions owing to their unique structure [19]. In the current work, with the purpose of combining GE possessing excellent TC with ILs having good thermophysical properties along with the virtue of making GE functionalized, GE has been dispersed into the IL 1-hexyl-3-methylimidazolium tetrafluoroborate ([HMIM]BF_4_) without using any surfactant to prepare novel GE-based Ionanofluids for the first time. The thermophysical properties of the GE-dispersed Ionanofluids were investigated together with those of the Ionanofluids containing MWCNTs for comparison purpose.

## Methods

### Chemicals and materials

MWCNTs and graphite were purchased from Nanjing XFNano Material Tech Co., Ltd. (China); H_2_SO_4_, HNO_3_, and KMnO_4_, from Alfa Aesar (Ward Hill, MA, USA). [HMIM]BF_4_ (CAS number, 244193-50-8) was provided by Lanzhou Institute of Chemical Physics, Chinese Academy of Sciences. Other reagents such as H_2_O_2_ and N_2_H_4_·H_2_O were used as received.

### Synthesis of GE nanosheets

Graphite oxide (GO) was synthesized using Hummers' method [20]. Graphite powder (2.0 g) was put into cold (4 °C) concentrated H_2_SO_4_ (46 mL) followed by gradually adding 6.0-g KMnO_4_ under stirring for 2 h while the temperature of the mixture was kept at below 10 °C. After stirring the mixture at 35 °C for 30 min, 92 mL of deionized (DI) water was slowly added into the system to keep the temperature of the mixture at 98 °C for 15 min. Then, the mixture was further diluted using approximately 300-mL DI water. After that, 15-mL H_2_O_2_ (30%) was added to the mixture to reduce the residual KMnO_4_ until the color of the mixture changed into brilliant yellow. Finally, the mixture was filtered and washed with 5% of HCl aqueous solution to remove metal ions followed by washing with 1.0 L of DI water to remove the acid. The obtained solid was dried at 60 °C for 24 h. For further purification, the as-obtained GO was re-dispersed in DI water and then was dialyzed for 1 week to remove residual salts and acids.

Prepared GO powder (100 mg) was added to 100-ml water. After being ultrasonically dispersed for 1 h, 1-g hydrazine hydrate was added to the mixture followed by being refluxed for 24 h to reduce graphene oxide to GE nanosheets. The solid product was isolated by centrifugation, washed with distilled water and ethanol for three times, and finally dried at 60 °C in a vacuum oven for 24 h to remove residual solvent. 

### Preparation of ionanofluids based on ILs

**Figure 1:**
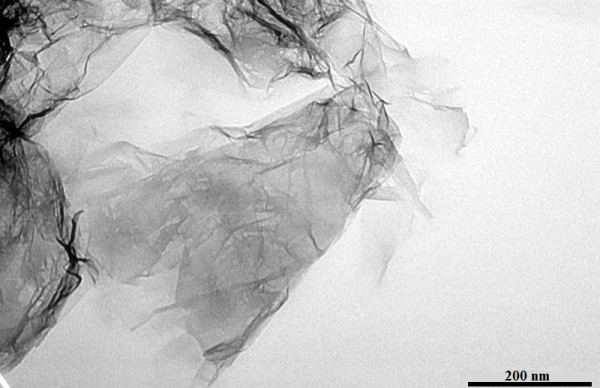
**Digital photograph of (a) pure [HMIM]BF**_**4**_**, (b) 0.03% of GE/[HMIM]BF**_**4**_**, and (c) 0.03% of MWCNT/[HMIM]BF**_**4**_**.**

MWCNTs and GE were dispersed into [HMIM]BF_4_ using a 100-W, 40-kHz ultrasonicator for 8 h. Then, the mixtures were sonicated for 2 min using a 25-W Ultrusonic Cell Disrupter System (JYD 900, Shanghai Zhisun Equipment Co., Ltd, China). Figure 1 displays the digital photograph of the graphene and MWCNT-dispersed nanofluids, respectively. In this paper, we prepared Ionanofluids at very low weight percentage of 0.03% and 0.06%; when the weight percentage goes up to 0.09%, the Ionanofluids are not stable and they will coagulated in a couple of hours.

**Figure 2:**
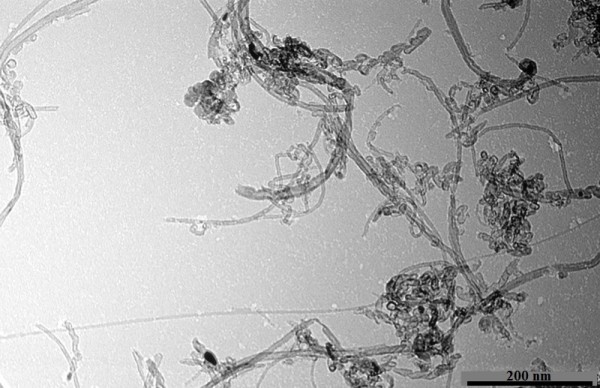
TEM image of pristine GE.

**Figure 3:**
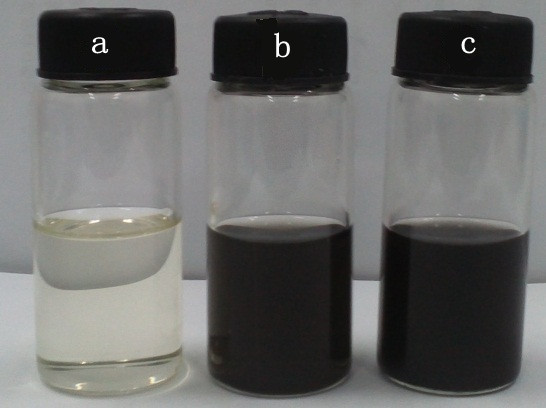
TEM image of MWCNTs.

### Characterization and measurements

TEM images were obtained on a PHILIPS TECNAI 10 electron microscope (FEI Corporation, Hillsboro, OR, USA) at an accelerating voltage of 100 kV. The TEM samples were prepared by dispersing the powder products in alcohol by ultrasonic treatment followed by dropping the suspension onto a holey carbon film supported on a copper grid and drying it in air. Dispersion and stability of these ionanofluds were observed by a light microscope (LEICA, DM 2500P, Leica Microsystems Ltd., Milton Keynes, UK) at same magnification (×500).

TC of the samples were measured at the temperatures ranging from 25 °C to 65 °C using a thermal constants analyzer (Hot Disk TPS 2500 S, Hot Disk AB, Gothenburg, Sweden). In order to precisely control the temperature, a cyclic silicone oil bath was applied. After every increase in temperature, the samples were equilibrated for at least 5 min before measurements. The TC measurements were repeated several times, and the average values were calculated for use in this paper.

The specific heat of the samples were evaluated with a differential scanning calorimeter (DSC Q20, TA Instruments, New Castle, DE, USA) using the sapphire method. The temperature was kept at 0 °C for 5 min then ramped to 80 °C at the increasing rate of 10 °C ·min^−1^ followed by keeping for another 5 min. We have checked the accuracy of the measurements by measuring the specific heat of DI water between 20 °C and 85 °C and found deviations less than 0.98%, with an average deviation of 0.418%.

The viscosities of the samples were measured by a viscometer (DV-2 + PRO, Shanghai Nirun Intelligent Technology Co., Ltd, Yangpu, Shanghai, China) at a revolution rate of 100 rpm. Each sample was measured at the temperatures ranging from 25 °C to 75 °C.

## Results and discussion

### TEM analysis

The morphology and structure of the pristine and GE and MWCNT were observed by TEM, which were observed again after all the experiments, as shown in Figures 2 and 3. The observations from Figures 2 and 3 revealed that the received pristine MWCNT was not only aggregated, but entangled, whereas the GE we prepared was relatively well dispersed and stretched. The obtained Ionanofluids are black and can keep stable for a long time. It is suggested that GE and MWCNTs have good dispersity in [HMIM]BF_4_, which is probably attributed to that GE and MWCNTs can be functionalized by [HMIM]BF_4_.

### Stability and dispersion of ionanofluids

**Figure 4:**
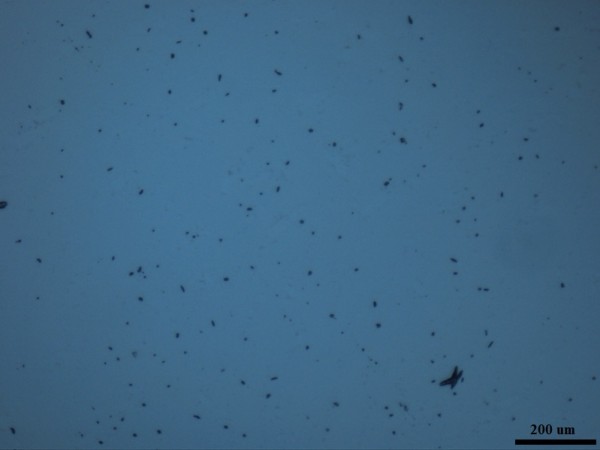
**Optical image of graphene/[HMIM]BF**_**4**_**Ionanofluid (0.03 wt.%).**

**Figure 5:**
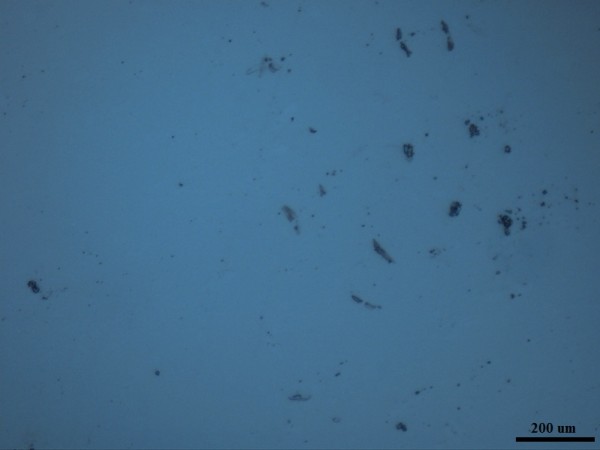
**Optical image of MWCNT/[HMIM]BF**_**4**_**Ionanofluid (0.03 wt.%).**

Optical images of these Ionanofluids were taken by putting a drop of fluid on a cleaned glass and observing the dispersion of particles in the base fluids. Figures 4 and 5 show that the MWCNT is not well dispersed in [HMIM]BF_4_ as the graphene does. The nonhomogeneity of MWCNT particles is visible in Figure 3b, while Figure 3a shows uniform dispersion and distribution of graphene particles in [HMIM]BF_4_. Compared to MWCNT/[HMIM]BF_4_ Ionanofluid, graphene/[HMIM]BF_4_ Ionanofluid is more homogeneous and stable.

### Thermal conductivity of ionanofluids

**Figure 6:**
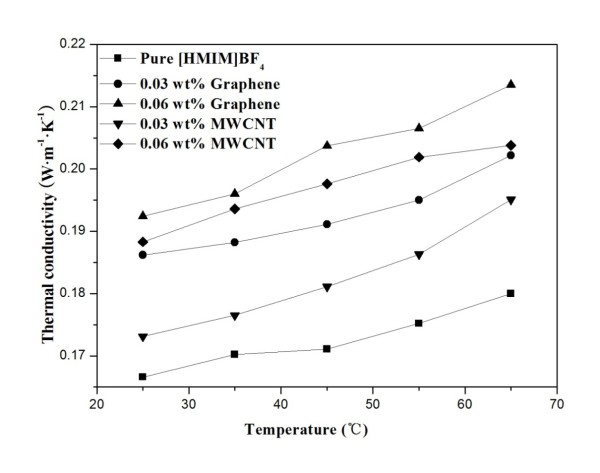
TC enhancement as a function of temperature.

TC of the obtained Ionanofluids was measured at different temperatures by the transient plane source (TPS) method, which is an advanced technique evolved from the hot wire method by Hot Disk AB. As shown in Figure 6, the TC of the Ionanofluid containing 0.03% of GE increases from 0.1862 to 0.2022 W·m^−1^·K^−1^ as the temperature increases from 25 °C to 65 °C and accordingly increases from 0.1924 to 0.2135 W·m^−1^·K^−1^ when the GE loading is 0.06%. In this paper, *k* and *k*_0_ refer to the TC of the Ionanofluid and the pure ionic fluid, respectively, and (*k* − *k*_0_)/*k*_0_ is defined as the TC enhancement ratio. The TC enhancement ratios of the Ionanofluid containing GE of 0.03% range from 11.8% to 12.3% as the tested temperature varies from 25 °C to 65 °C and accordingly increases from 15.5% to 18.6% as the GE loading is increased to 0.06%. It is indicated that the TC enhancement ratio of the GE-dispersed Ionanofluids increases with the mass fraction of GE. The remarkable TC enhancement ratio of more than 10% is achieved by the Ionanofluid containing GE with the mass fraction of as low as 0.03%, implying that GE is a good nanoadditive for the nanofluids based on ILs. For the Ionanofluids containing MWCNTs, their TC enhancement ratios range from 3.9% to 8.4% as the tested temperature varies from 25 °C to 65 °C and accordingly increases to 13.0% and 13.2% as the MWCNT loading is increased to 0.06%. It is revealed that the TC enhancement ratios of the MWCNTs-based Ionanofluids are less than those of the GE-based Ionanofluids at the same nanoadditive loading, which is probably due to the extraordinary high thermal conductivity of GE and Brownian motion of nanoparticles at the molecular and nanoscale levels [21]. Significantly, the TC enhancement ratios of all the Ionanofluids are less than 20%, consistent with the results obtained from the benchmark study on the TC of nanofluids [22]. No anomalous TC enhancement is achieved by the obtained nanofluids, in which their TC values have been measured by the TPS method other than the hot wire method commonly used in the previous work.

### Viscosity studies of ionanofluids

**Figure 7:**
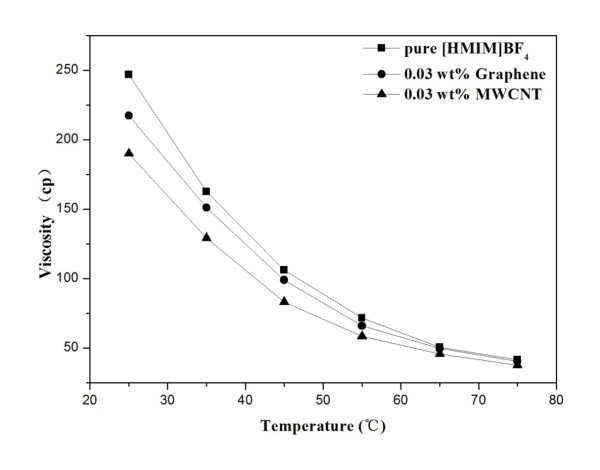
Viscosity as a function of temperature.

The viscosities of pure [HMIM]BF_4_ and the GE- and MWCNT-dispersed Ionanofluids at the same nanoadditive loading of 0.03 wt.% were measured at different temperatures, respectively. As plotted in Figure 7, the viscosity of the Ionanofluid containing 0.03% of GE decreases from 217.4 to 40.6 cp as the temperature increases from 25 °C to 75 °C, indicating that the viscosity of the Ionanofluid can be dramatically reduced by increasing temperatures. Previous works also demonstrated the decrease of the viscosity of nanofluids against the increase of temperature [23-25]. However, according to previous reports, the viscosity of TiO_2_/water nanofluids [26] and Al_2_O_3_/water nanofluids [27] is found to be substantially higher than the values of pure water, alumina/propylene glycol nanofluids [28], and copper/ethylene glycol nanofluids [29], which are reported to exhibit higher viscosity than their base fluids. The viscosity increase of these nanofluids compared with their base fluids may restrict their application as heat transfer fluids. Conversely, in our experiments, the addition of GE or MWCNTs can slightly decrease the viscosity of the base fluid, consistent well with Baogang Wang's result, in which the F-MWCNTs/[Bmim][PF_6_ nanofluids show lower viscosity than pure [Bmim][PF_6_ especially under high shear rates [30]. This phenomenon could be attributed to the self-lubrication of GE and MWCNTs. Definitely, the Ionanofluids with lower viscosity and higher TC than their base fluids are what we expected to see.

### Specific heat study of ionaofluids

**Figure 8:**
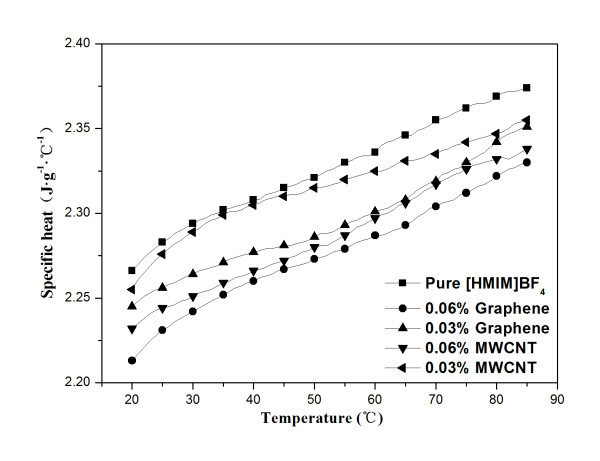
Specific heat as a function of temperature.

The specific heat of pure [HMIM]BF_4_ and the GE- and MWCNT-dispersed Ionanofluids were tested at the same nanoadditive loading of 0.03 and 0.06 wt.% at different temperatures. As shown in Figure 8, the specific heat of pure [HMIM]BF_4_ increases from 2.266 to 2.369 J·g^−1^·°C^−1^ as the temperature increases from 20 °C to 80 °C, indicating that the specific heat of the IL increases with the tested temperature, and the same trend was found in Ionanofluids too. It can be seen that the specific heat of the GE-dispersed Ionanofluid is lower than that of the MWCNT-dispersed one at the same loading. Moreover, the specific heat capacities of these Ionanofluids are lower than that of the neat IL at the same temperature, which has been also observed for the carbon black-based Ionanofluid. According to Bridges' research, the specific heat of pure [C_4_mmim][NTf_2_ decreases from 1.53 to 1.34 J·g^−1^·°C^−1^ after adding carbon black of 0.5 wt.%, which means the nanoparticles cause a decrease of specific heat for more than 12% compared with pure IL at a very low loading of 0.5 wt.%. The authors point out that the decrease in specific heat for the Ionanofluid may be due to the increased thermal conductivity of the Ionanofluid [11]. In our experiments, the specific heat of the Ionanofluid containing 0.03% of graphene decreases by 0.93% compared with the pure [HMIM]BF_4_ at 20 °C and by 1.14% at 80 °C, which cannot be explained by traditional theory [31]. In fact, a theoretical model for the specific heat of nanofluids should take other factors into account, such as thermal conductivity, thermal diffusivity, size effects, the interactions of the surface atoms of nanoparticles and the surroundings, and pH [32,33]. In this paper, the decrease in specific specific heat of nanofluids may be attributed to that the specific heat of the nanoadditives are lower than that of the base fluid and the increased TC of the Ionanofluids.

## Conclusions

GE and MWCNTs can be dispersed into [HMIM]BF_4_ without using any surfactant. The GE-dispersed Ionanofluids are more homogeneous and stable than those containing MWCNTs at the same nanoadditive loading. The remarkable TC enhancement ratio of more than 10% is achieved by the Ionanofluid containing GE with the mass fraction of as low as 0.03%. The TC enhancement ratios of the GE-based Ionanofluids are larger than those of the MWCNT-based Ionanofluids at the same nanoadditive loading. No anomalous TC enhancement is achieved by all the GE- and MWCNT-dispersed Ionanofluids. The Ionanofluids exhibit lower viscosity than their base fluids, which is beneficial for their application as heat transfer fluids. The specific heat of the GE- and MWCNT-dispersed Ionanofluids is very close to that of the pure IL. Ionanofluids containing graphene are a new class of heat transfer fluids which exhibit fascinating thermophysical properties compared to the base ionic liquids; they have the potential applications from refrigeration systems at the low temperature end to solar energy collection at high temperatures owing to their unique characteristics of a wide range of liquid temperature, low vapor pressure, and high thermal stability. The further experimental research on the thermal and optical properties of Ionanofluids containing graphene at high temperature will be conducted in our future work.

## Abbreviations

GE, Graphene; GO, Graphite oxide; Ionanofluids, Ionic liquid-based nanofluids; ILs, Ionic liquids; MWCNTs, Multi-walled carbon nanotubes; TC, Thermal conductivity; TEM, Transmission electron microscopy.

## Competing interests

The authors declare that they have no competing interests.

## Authors’ contributions

FW designed and conducted the experiments, carried out the experimental analyses, and drafted the manuscript. JS and LH prepared the GO and GE. ZZ, XF, and WM conceived the study, participated in its design and coordination, wrote the introduction, and modified the manuscript. All authors read and approved the final manuscript.

## Authors’ information

FW received his Bachelor in Material Chemistry from Wuhan University of Technology, China in 2010, and now, he is a postgraduate student at South China University of Technology. His research interests include heat transfer enhancement, nanofluid, and advanced materials. LH received her Bachelor in Chemical Engineering and Technology from Central South University, China in 2010, and now, she is a postgraduate student at South China University of Technology. Her research interests are optical functional materials. XF received her Bachelor in Organic Chemical Engineering from Chengdu University of Science and Technology in 1990 and PhD in Chemical Engineering from South China University of Technology in 2002. She did her postdoctoral research at National Institute of Advanced Industrial Science and Technology, Japan. She is now a professor at South China University of Technology. Her research interests include phase change and solar cell materials. ZZ received his bachelor degree from Sichuan University in 1990 and PhD from South China University of Technology in 1996. He is now a professor at South China University of Technology and a research leader at the Key Laboratory of Enhanced Heat Transfer and Energy Conservation of the Ministry of Education, China. His research interests include heat transfer enhancement, composite phase change material, and nanofluid. WM received his PhD from South China University of Technology in 1996. He is now a professor at South China University of Technology. His research interests include composite material and nano-technology.
